# All-laser-micromachining of ridge waveguides in LiNbO_3_ crystal for mid-infrared band applications

**DOI:** 10.1038/s41598-017-07587-w

**Published:** 2017-08-01

**Authors:** Lingqi Li, Weijie Nie, Ziqi Li, Qingming Lu, Carolina Romero, Javier R. Vázquez de Aldana, Feng Chen

**Affiliations:** 1 School of Physics, State Key Laboratory of Crystal Materials, Shandong University, Jinan, 250100 China; 2 School of Chemistry and Chemical Engineering, Shandong University, Jinan, 250100 China; 3 Grupo de Investigación en Aplicaciones del Láser y Fotónica, Departamento de Física Aplicada, University of Salamanca, Salamanca, 37008 Spain

## Abstract

The femtosecond laser micromachining of transparent optical materials offers a powerful and feasible solution to fabricate versatile photonic components towards diverse applications. In this work, we report on a new design and fabrication of ridge waveguides in LiNbO_3_ crystal operating at the mid-infrared (MIR) band by all-femtosecond-laser microfabrication. The ridges consist of laser-ablated sidewalls and laser-written bottom low-index cladding tracks, which are constructed for horizontal and longitudinal light confinement, respectively. The ridge waveguides are found to support good guidance at wavelength of 4 μm. By applying this configuration, Y-branch waveguiding structures (1 × 2 beam splitters) have been produced, which reach splitting ratios of ∼1:1 at 4 μm. This work paves a simple and feasible way to construct novel ridge waveguide devices in dielectrics through all-femtosecond-laser micro-processing.

## Introduction

As the basic components in integrated photonics, optical waveguides could confine light propagation within small volumes with dimensions of micrometric or sub-micrometric scales, in which higher optical intra-cavity intensities could be achieved compared with bulk materials^1^. Therefore, enhanced optical properties, e.g., lasing performances and nonlinear responses^2–4^, could be realized in optical waveguides. Particularly, compared with planar waveguide, the ridge waveguides with advantage of stronger spatial confinement of light propagation are particularly desirable for the construction of intricate photonic devices^5^. As a result, some performances of ridge waveguides are intriguing, such as the enhanced efficiency of nonlinear devices^3, 6^ and reduced thresholds in integrated lasers^7^. Additionally, the Y-branch waveguides, an essential element for beam splitting or interferometers, has been widely used for electro-optical modulations, optical communications, or integrated optical chips^8, 9^. In addition to silicon, transparent dielectrics are also favorite medium for waveguide devices. Various techniques have been utilized to fabricate diverse guiding structures in transparent optical materials^10, 11^. Generally, in order to fabricate the 2D ridge waveguides, it is required ridge construction on base of planar waveguide layer. For example, in dielectric crystals, a planar waveguide may be formed by ion irradiation or ion implantation in the first step, and second processing with other techniques, such as wet or dry etching, ion-beam enhanced etching, diamond blade dicing, and laser ablation, on the planar waveguide may be implemented to remove the selected parts of the planar waveguide surface, thus constructing the ridge waveguides^12–16^. Nevertheless, these traditional fabrication solutions require combination of at least two-step processing of diverse techniques. In practice, more convenient and flexible fabrication method of ridge waveguide is desired.

Different from previous works, the ridge structure in this work is fully fabricated by femtosecond (fs) laser micromachining without the combination of other methods. Femtosecond laser micromachining, as one of the most efficient methods, has been widely applied to implement three-dimensional one-step micro-processing in diverse transparent materials for a great variety of applications^17–22^ since the pioneering work by Davis *et al*. in 1996^23^. During the processing of fs-laser inscription, the high intensity femtosecond laser is focused at a sample spot at certain depth, resulting in a localized micro-modification of the materials with a refractive index change (negative or positive) through nonlinear absorption processes followed by strong-field ionization and avalanche ionization^24^. In the dielectric crystals, the femtosecond laser pulses usually induce negative changes (Δ*n* < 0) of the refractive index due to the localized lattice expansion at the focusing volume (i.e., inside the laser-induced tracks), which could be used as low index cladding for waveguide formation (the waveguide cores locate in the regions surrounded by these tracks of Δ*n* < 0 or regions between the tracks due to the stress-induced effect)^25, 26^. The high intensity pulses of fs-lasers not only produce material damage in the focal volume or surroundings, but can be also used to etch crystals in selected regions through the ultrafast ablation mechanism (Coulomb explosion)^7, 27^. In this work, the novel ridge waveguides are fabricated via all-laser-micromachining: the waveguide core is with lateral confinement of laser-ablated grooves and longitudinal restriction of laser-written bottom low-index cladding tracks. Compared to the ordinary ridge waveguide based on planar waveguide, the fabrication of this geometry of ridge waveguides has shown the superiority of laser-micromachining, such as wide applicability of materials, negligible effect of thermal-diffusion, and ability for maskless 3D processing^18^.

The mid-infrared (MIR), typically ranging from 2.5 to 10 μm wavelength, is a strategically important spectral band for a number of applications such as biochemical detection, environmental monitoring, and free-space communication^28^. As one of the widely used multifunctional crystals, lithium niobate (LiNbO_3_) is an ideal candidate due to its unique combination of superior properties such as electro-optic, acousto-optic and piezoelectric properties^29–31^. Particularly, the transparency of LiNbO_3_ crystals ranges from 420 nm to 5200 nm, covering bands from violet till MIR regime. Recently, the LiNbO_3_ crystal has attracted tremendous investigations in MIR band applications^32–34^. Benefiting from the above mentioned advantages, the LiNbO_3_ crystal is promising to achieve efficient combination of excellent nonlinear bulk performance and the ridge structures in MIR band. So far, femtosecond-laser micromachined waveguides of Type I direct-write waveguides, Type II stress-induced waveguides^35–38^, and depressed cladding waveguides geometries^25^ have been realized in LiNbO_3_ crystals. However, ridge waveguides in LiNbO_3_ crystal via all-laser-micromachining and their applications to the MIR regime have not been reported. In this work, the novel ridge waveguide and Y-branch structures have been achieved in LiNbO_3_ crystal via all-laser-micromachining, which pave a promising way for a wide variety of applications in many disciplines. The guiding properties at MIR wavelength of 4 μm has been measured and discussed in detail. Our work demonstrated a flexible and convenient manner to construct novel ridge geometries in dielectrics via all-femtosecond-laser micro-processing for photonic applications.

## Results

**Figure 1:**
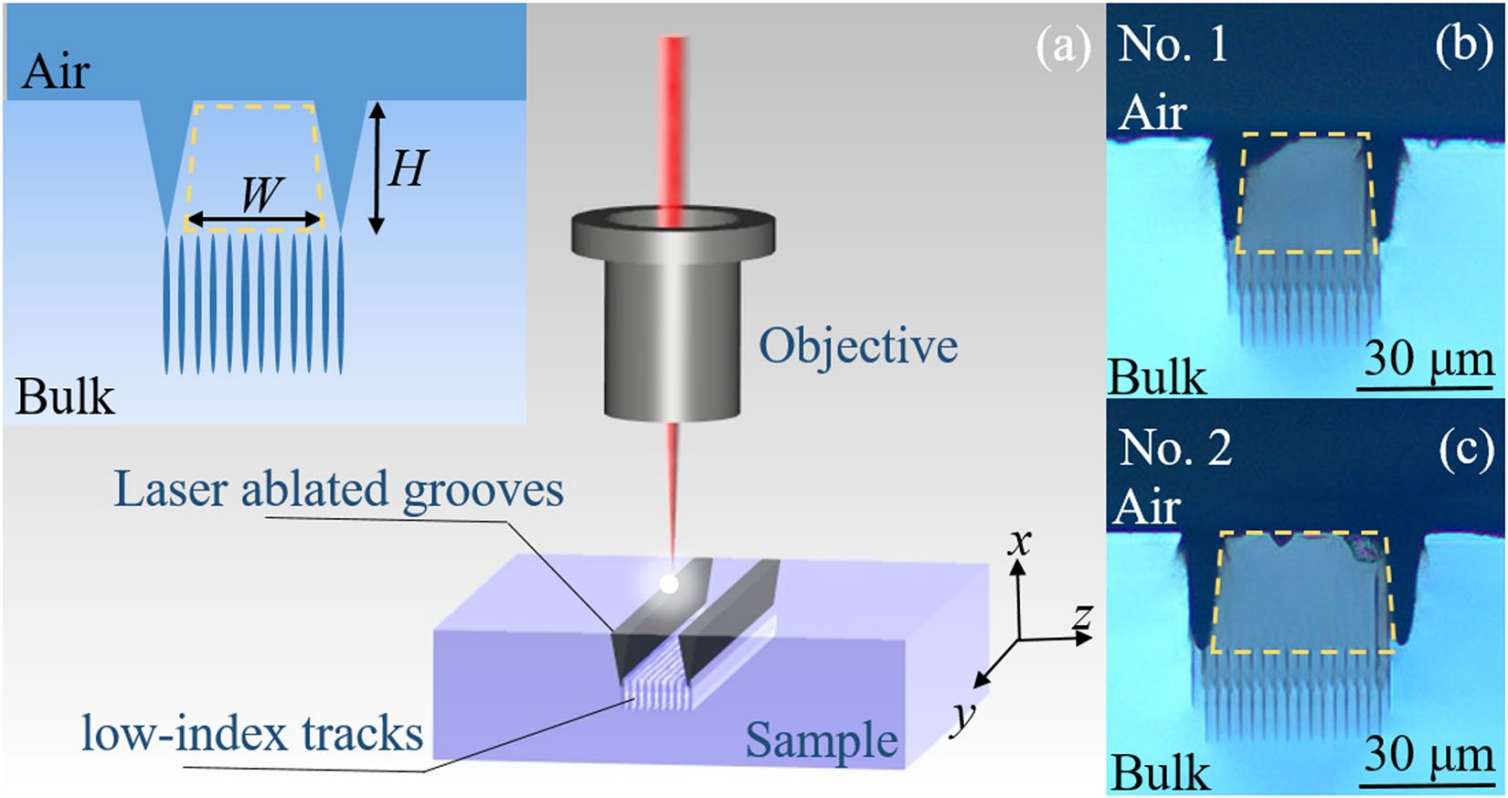
The fabrication of ridge waveguides. (**a**) Schematic plot of the fabrication process with the femtosecond laser. The inset picture is the schematic diagram of the ridge configuration. Optical microscope image of the cross section of ridge waveguides No. 1. (**b**) (top) and No. 2 (**c**) (bottom). The yellow dashed frames indicate the spatial locations of the ridges.

The novel ridge waveguides in LiNbO_3_ crystal are fabricated by all-laser-micromachining technique. Figure 1(a) depicts the process of the femtosecond laser micromachining. Focused fs-laser pulse produces localized modification in the focal volume forming the laser-written low-index cladding tracks and laser-ablated grooves, which depends on the parameters of the applied fs-laser pulse such as pulsed energy, repetition rate, pulse duration, scanning speed and beam polarization. The inset picture of Fig. 1(a) is the schematic diagram of the ridge configuration, *W* denotes the bottom-width of the ridges, and *H* ~ 25 μm is the ablated depth. Figure 1(b) and (c) demonstrate the optical microscope images of the cross-sectional of ridge waveguides No. 1 (*W* = 30 μm) and No. 2 (*W* = 50 μm), in which the ridge outlines can be seen clearly. The core of ridges, as identified by the yellow dashed frame, is located in the regions surrounded by the fs-laser inscribed tracks and fs-laser ablated grooves.

**Figure 2:**
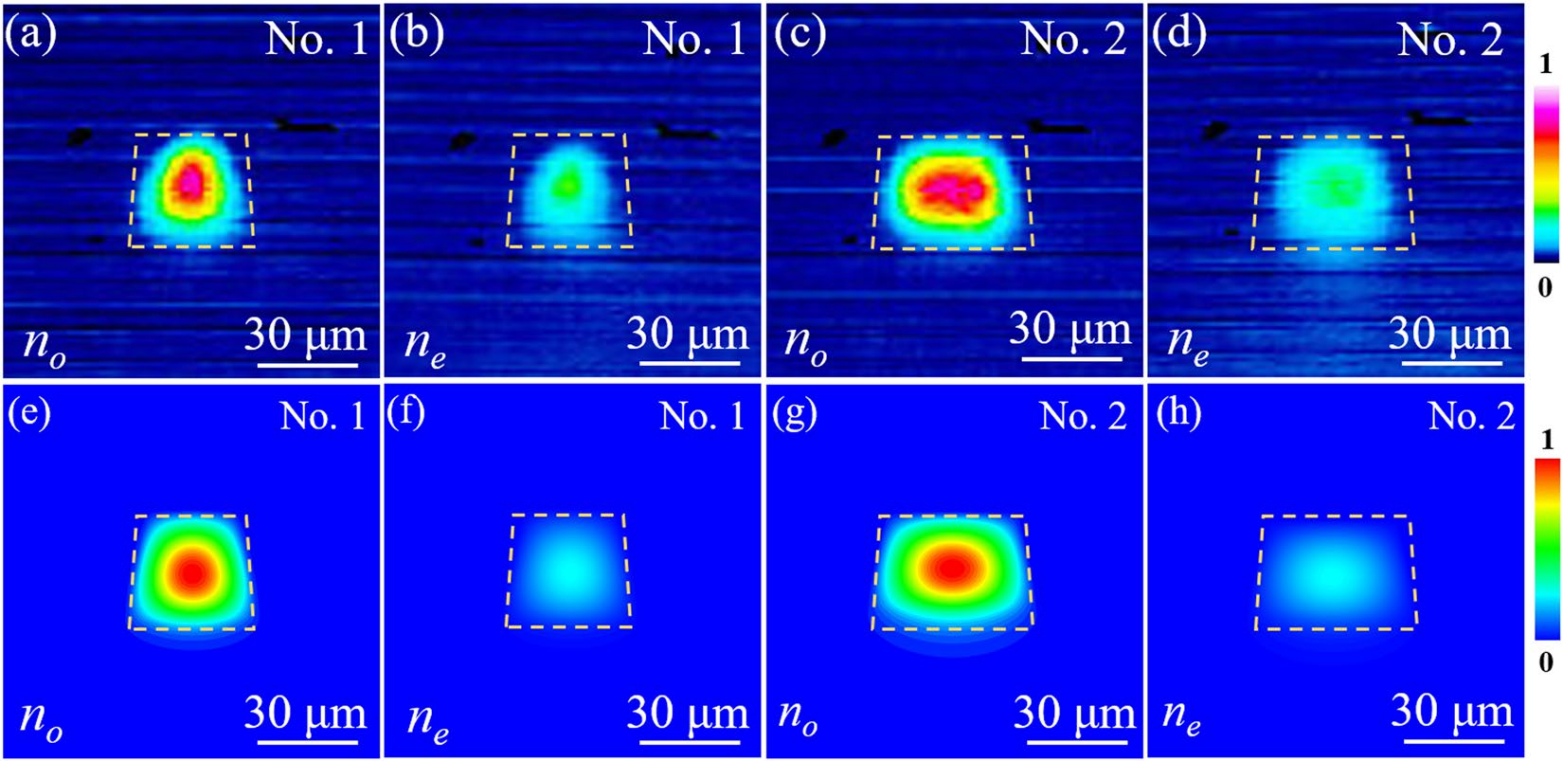
Guiding properties of ridge waveguides. Measured near-field modal distributions of LiNbO_3_ ridge waveguides at 4 μm. (**a**) and (**b**) No. 1, (**c**) and (**d**) No. 2, along *n*_*o*_ (left) and *n*_*e*_ (right) polarization. Calculated near-field modal distributions (**e**) and (**f**) No. 1, (**g**) and (**h**) No. 2, along *n*_*o*_ (left) and *n*_*e*_ (right) polarization at 4 μm.

To experimentally investigate the guiding properties of the ridge waveguides along 10-mm-long *y*-axis at MIR band, an end-face coupling arrangement at wavelength of 4 μm with a linearly polarized laser is employed. Figure 2(a)–(d) illustrate the measured near-field intensity distribution of the waveguides No. 1 and No. 2 along *n*_*o*_ and *n*_*e*_ polarizations at wavelength of 4 μm, respectively. As one can see, both waveguides (No. 1 and No. 2) have shown better guidance along the *n*_*o*_ polarization at MIR wavelength range that those along the *n*_*e*_ polarization, which is in good agreement with the depressed cladding waveguides in LiNbO_3_ crystal produced by femtosecond laser micromachining^25^.

**Figure 3:**
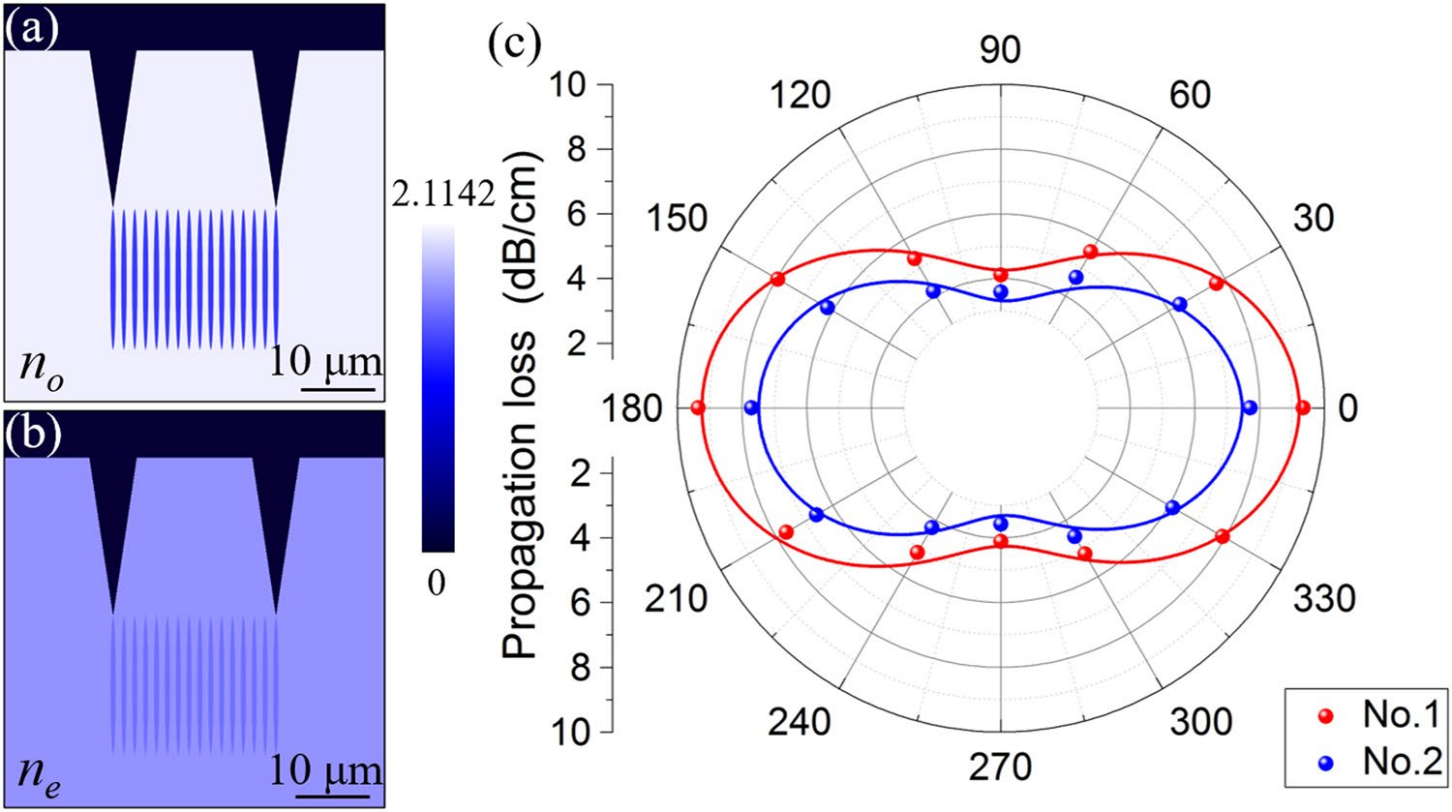
Reconstructed refractive index profile and polarization properties of ridge waveguides. Reconstructed refractive index profile of *n*_*o*_ (**a**) and *n*_*e*_ (**b**) of No.1. (**c**) Polarization images of the propagation loss of ridges No. 1 and No. 2 at 4 μm.

In addition, we calculate the modal profiles of the waveguides numerically based on the estimation of the laser-induced refractive index changes. By assuming a step-index profile, the maximum refractive index modification between the waveguide core and substrate can be roughly estimated with the Eq. 1

1 $${\rm{\Delta }}{n}_{o,e}\approx \frac{{\sin }^{2}{\theta }_{m}}{2{n}_{o,e}}$$

where *θ*_*m*_ is the maximum incident angular deflection at which no change of the transmitted power occurred, *n*_*o*_ and *n*_*e*_ are the refractive index of LiNbO_3_ at 4 μm. In this work, the calculated maximum refractive index change for waveguides No. 1 and No. 2 at 4 μm are 4.8 × 10^−3^ and 5.2 × 10^−3^ along the *n*_*o*_ polarization, respectively. However, the values of the *n*_*e*_ polarization are 1.3 × 10^−3^ and 2.1 × 10^−3^, which are much smaller than those along the *n*_*o*_ polarization. The smaller refractive index alternations along the *n*_*e*_ polarization may also cause larger propagation loss along the *n*_*e*_ polarization. With the obtained refractive index changes, the spatial distributions of the index have been reconstructed (Fig. 3(a) (*n*_*o*_) and Fig. 3(b) (*n*_*e*_)). According to these 2D refractive index profiles, the near-field intensity profiles are simulated by the FD-BPM algorithm (Rsoft^®^Beam PROP)^39^, which is based on the finite difference beam propagation method (FD-BPM)^40^. Figure 2(e)–(f) illustrate the calculated modal distributions of the waveguides along both *n*_*o*_ and *n*_*e*_ polarizations at 4 μm, showing a good accordance with the experimental data.

**Figure 4:**
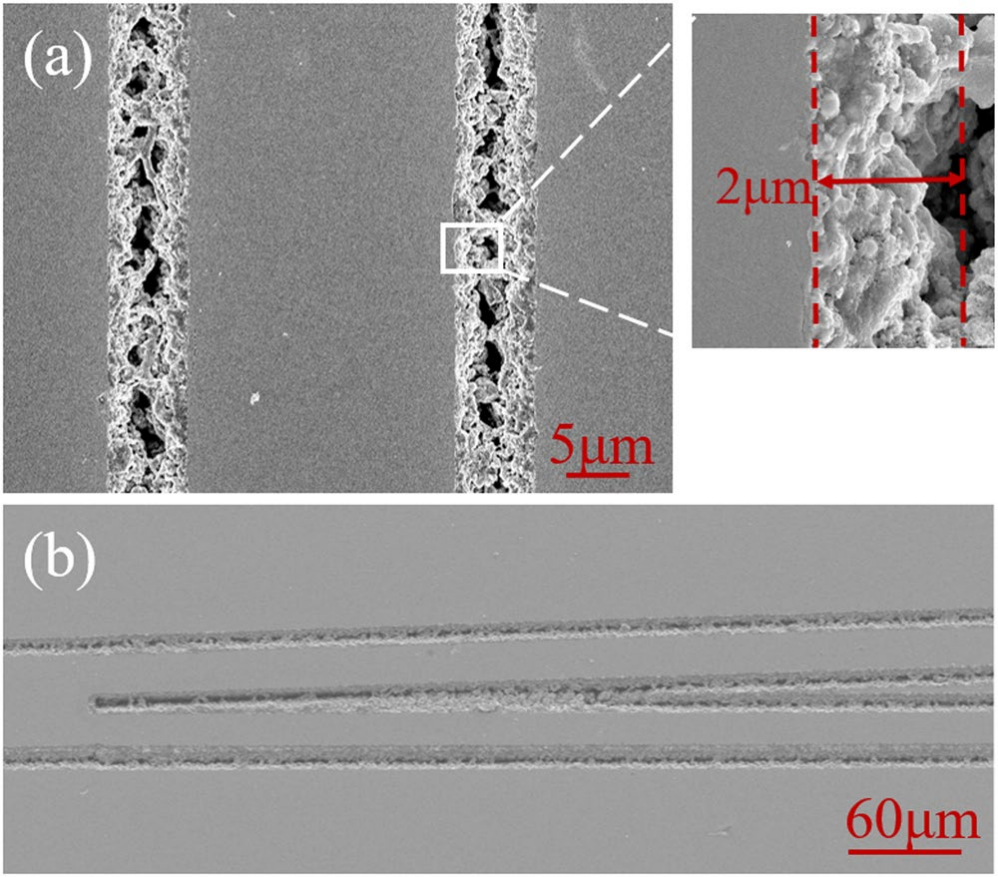
The topographic SEM image of ridge waveguides. (**a**) The SEM image of the ridge waveguide surface. The inset picture show the sidewalls of ridge waveguide. (**b**) The SEM image of the intersection of Y-branch.

Based on the end-face coupling arrangement, the propagation losses of the ridge waveguide of LiNbO_3_ were measured. The propagation loss can be calculated by using the Eq. 2 ^41^:

2 $$\alpha =\frac{-10}{L}{\mathrm{log}}_{10}[\frac{{P}_{{\rm{out}}}}{\eta {P}_{{\rm{in}}}{(1-R)}^{2}}]$$

where *P*_*in*_ is the in-coupled light power and *P*_*out*_ is the output light power. The length of light propagating in the sample is written as *L*. *R* is the reflectance, and we calculate *R*_*o*_ ~ 0.87 and *R*_*e*_ ~ 0.89 at 4 μm. Considering the mismatch coefficient *η* which can be calculated by an overlap integral between both the launched Gaussian field profiles and the modal profiles of the structure. The mismatch coefficient *η* can be expressed as^42, 43^

3 $$\eta =\int {\varphi }_{in}^{\ast }(x)\varphi (x,z)dx$$

where ${\varphi }_{in}(x)=\sum _{m}{c}_{m}{\varphi }_{m}(x)$ is the incident field, which is assumed as the Gaussian field. $\varphi (x,z)=\sum _{{\rm{m}}}{c}_{m}{\varphi }_{m}(x){e}^{i\beta z}$ is the propagating modal profiles of the structure and *β* is the propagation constant. The values of the mismatch coefficient have been calculated by FD-BPM algorithm (Rsoft^®^ Beam PROP), which are ~0.53 and 0.47 for waveguides No. 1 and No. 2, respectively. Finally, we can approximately calculate the propagation losses (listed in Table 1.) of the ridge waveguides in LiNbO_3_. The higher attenuation for ridge waveguide should be partly attributed to the non-perfect side-walls fabricated by the fs-laser. It is clearly that the propagation loss decreases with the increase of the width of ridge waveguides from 30 μm to 50 μm. The phenomenon, we believe, is caused by the lateral roughness of the ridges and the rough sidewalls affect the light propagation more strongly for narrower waveguide. In order to estimate the roughness of the sidewalls, we used scanning electron microscopy (SEM) to obtain the surface topographic image of the ridge waveguide (see Fig. 4(a)). As one can see, the roughness is estimated to be ~2.0 μm as the insert shows. One can expect the reduction of the roughness of the side walls by using post-ablation treatment (such as ion-beam sputtering)^44^, multiscan (instead of single-scan) of fs-laser ablation^45^ and wet etching^46^, which will reduce the propagation losses to a certain extent. In order to achieve thorough information of the polarization effects for propagating properties, the all-angle light transmission along the transverse plane has been measured in waveguides No. 1 and No. 2, as shown in Fig. 3(c). The minimum propagation loss appears when polarization angles are 90° and 270°, which is along the *n*_*o*_ polarization. The maximum propagation loss occurs when polarization angles are 0° and 180° (*n*_*e*_ polarization).

**Table 1:** Propagation losses α (dB/cm) of the LiNbO_3_ ridge waveguides at 4 μm.

	No. 1	No. 2	Y-branch
*n*_*o*_	4.61	3.58	4.28
*n*_*e*_	9.35	7.71	12.09

**Figure 5:**
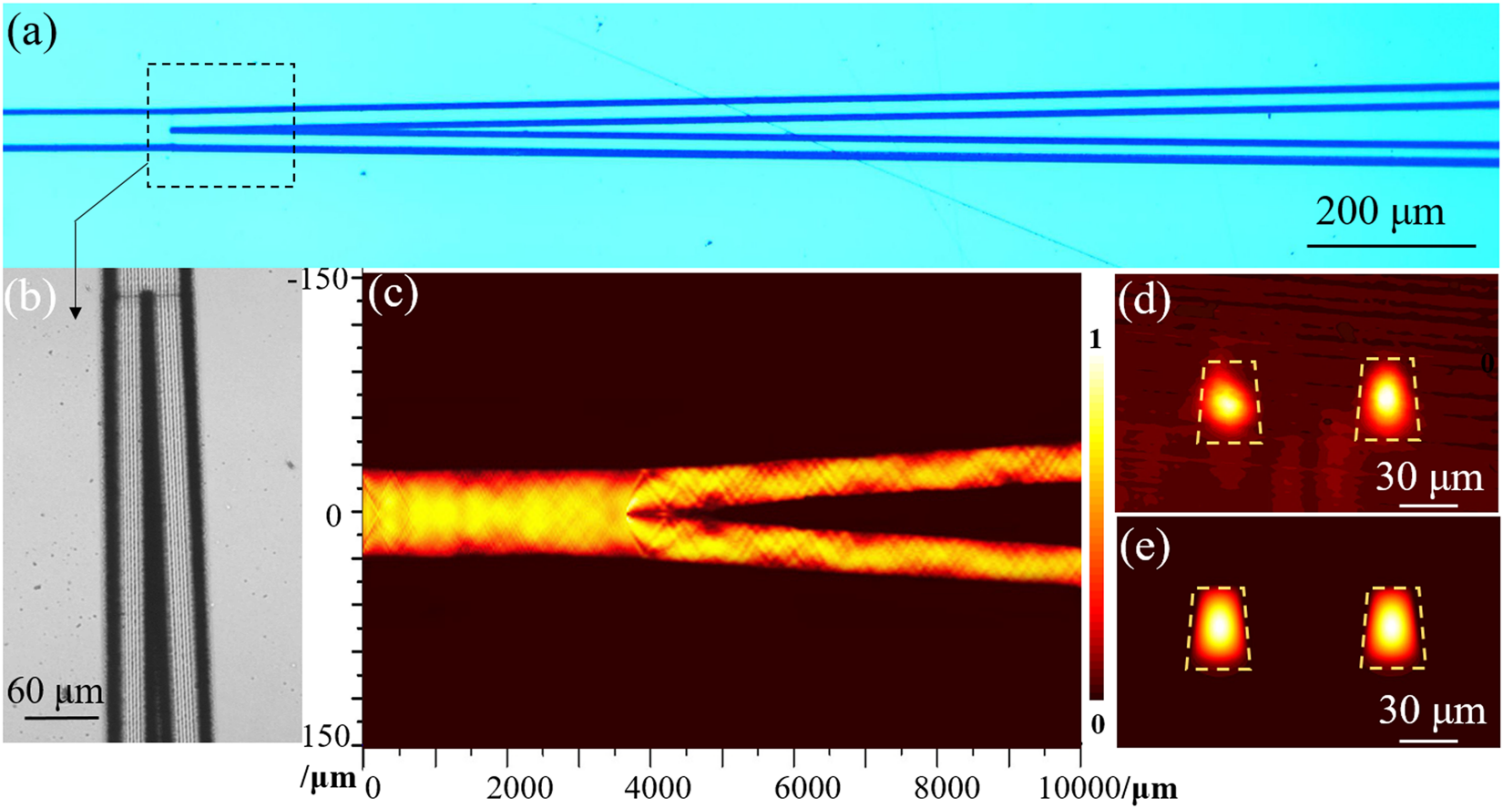
Optical microscope image and guiding properties of Y-branch waveguides. (**a**) The optical microscope image of the top view of the Y-branch. (**b**) The optical microscope image of the splitting point of the Y-branch. (**c**) The top view of simulated light propagating on the YZ-plane at 4 μm. (**d**) Measured modal profile of the cross-section of Y-branch at 4 μm. (**e**) Simulated modal profile of the cross-section of Y-branch at 4 μm.

By applying this novel ridge configuration, Y-branch structure (1 × 2 beam splitters) has successfully produced. The Y-branch structure waveguide is designed with a straight 3.8 mm length input arm (60 μm width), and two output arms (30 μm width) with the divergence of 0.46°, which corresponds to the lateral separation of 50 μm. Figure 5(a) depicts the top view of the Y-branch waveguide, the splitting point of the Y-branch structure can be clearly seen in Fig. 5(b), the darker line around splitting point is produced by the irradiation with a larger amount of pulses as it is a stopping and starting point for the stage motion. Figure 4(b) exhibits the topographic SEM images of the intersection of Y-branch structure. As expected, the good guiding performance along *n*_*o*_ polarization of the straight waveguides has also been found in the Y-branch splitter (Fig. 5(d)). Based on the calculated maximum refractive index change of 5.0 × 10^−3^ along *n*_*o*_ polarization, the near-field intensity profiles have been simulated. Figure 5(c) shows the top view of simulated light propagating on the YZ-plane at 4 μm. Moreover, the simulated Y-branch cross-sectional modal profile at 4 μm has also been depicted in Fig. 5(e), which is in good agreement with the experimental results (Fig. 5(d)). In addition, the propagation loss of the Y-branch is 4.28 dB/cm along *n*_*o*_ polarization at 4 μm. Particularly, the measured output splitting ratio for the two arms is 1:1.12, showing the excellent performance for the Y-branch splitter function.

## Discussion

The ridge structures consisting of laser-ablated sidewalls and laser-written bottom low-index cladding tracks have been designed and fabricated by in a LiNbO_3_ crystal wafer. The configuration of laser-ablated grooves and laser-written low-index tracks has been demonstrated to be an efficient way to build compact structures capable of efficient beam profile manipulation, including basic functions of beam splitting. In this work, the ridge waveguide and Y-branch structure have been achieved and implemented to the MIR regime in LiNbO_3_ crystal via all-laser-micromachining, which pave a promising way for a wide variety of applications in many disciplines. On the one hand, from the perspective of fabrication, our work shows the capability of fs-laser micro-fabrication to produce more complex waveguiding devices by integration of more designable elements in a single crystal chip, which would further enlarge the scope of promising applications related to this fabrication technique. Additionally, unlike the ordinary ridge waveguide based on planar waveguide, this technique has demonstrated the superiority of laser-micromachining, e.g., direct, rapid and mask-free features. On the other hand, by applying this ridge configuration, Y-branch structure (1 × 2 beam splitters) has successfully produced. The novel ridge structure in LiNbO_3_ with the lateral confinement of laser-ablation grooves and longitudinal restriction of laser-written low-index cladding tracks could be used for a wide variety of applications, such as electro-optical modulator, signal amplification, and frequency conversion. Finally, the exploration on the improvement of the sidewall quality to reduce the roughness is required in future work, which may be achieved by combining the suitable wet-etching (e.g., by HF acid). And one could extend this technique in other crystals to obtain waveguide devices for broader-scope applications of both scientific researches and human life. In conclusion, our work demonstrates a new ridge configuration by employing the all-laser-micromachining. Based on the new ridge waveguide, Y-branch structure has been implemented. This work paves a way to produce nonlinear optical devices for light guiding, beam splitting in dielectrics for various photonic applications at MIR regime.

## Methods

### Fabrication of ridge waveguides

The *x*-cut LiNbO_3_ crystal sample used in this work was cut into wafers with dimensions of 2 × 10 × 10 mm^3^ (*x* × *y* × *z*) and optically polished. To obtain the novel ridge waveguides and Y-splitters, a Ti:Sapphire amplified laser system (Spitfire, Spectra Physics) was utilized, in which linearly polarized pulses (120 fs duration, 796 nm central wavelength, 1 mJ maximum energy, and 1 kHz repetition rate) was delivered. In the first step to fabricate the low-index cladding tracks, the laser beam was focused at 20 μm below the largest surface (8 mm × 10 mm) by a 40 × microscope objective (N.A. = 0.65) with a pulse energy of ~0.32 µJ. The sample, located at a three-dimensional motorized stage with a spatial resolution of 0.2 μm. While the sample was scanned at a constant velocity of 500 μm/s along the 10-mm edge, a damage line was produced inside the sample. The procedure was repeated and several parallel low-index cladding tracks (3-μm lateral separation of adjacent parallel tracks) were inscribed at same depth of the sample, producing the longitudinal confinement of light propagation. Secondly, in order to get ablation grooves with depth of ~20 μm and with crater walls as vertical as possible, the laser beam was focused at the surface by a 10 × microscope objective (N.A. = 0.25). A larger pulse energy (four times larger than that on the first step) of 1.2 μJ, was set at a much slower scanning speed of 100 μm/s. Therefore, an ablation channel was formed. The fabrication procedure was repeated to produce an additional channel with lateral separation (the width of ridges) enough to equal the length of the bottom parallel low-index cladding tracks. With the lateral confinement of ablation grooves and longitudinal restriction of parallel low-index cladding tracks, the 10-mm long novel ridge waveguide was produced on the surface of the LiNbO_3_ crystal. The schematic plot of the experimental setup is depicted in Fig. 1(a).

### Characterization of Guidance

The cross-sectional microscope images of ridge waveguides were taken by using an optical microscope (Axio Imager, Carl Zeiss) operating in transmission mode. The SEM images were obtained using a field-emission scanning electron microscope (SU8010). The near-field modal distributions were investigated by employing a typical end-face arrangement. The linearly polarized laser (Daylight Solutions, Inc.), was focused with a MIR microscope objective lens (ZnSe, LFO-5-12-3.75, N.A. = 0.13) in one end-face of the waveguides. Afterwards, the modal profile at the output of the waveguide was imaged onto a MIR beam imaging camera (WinCamD, DataRay Inc.) by another same objective lens. Based on the above arrangement, the propagation losses were determined by directly measuring the light powers coupled into and out of the end-faces. The coupling efficiency was estimated by considering the overlap of the incident light beam and waveguide mode. The coupling and Fresnel reflection losses of the waveguide systems were calculated as well.
